# Antifungal Activity of Essential Oil and Plant-Derived Natural Compounds against *Aspergillus flavus*

**DOI:** 10.3390/antibiotics11121727

**Published:** 2022-12-01

**Authors:** Fei Tian, So Young Woo, Sang Yoo Lee, Su Been Park, Yaxin Zheng, Hyang Sook Chun

**Affiliations:** Food Toxicology Laboratory, School of Food Science and Technology, Chung-Ang University, Anseong 17546, Republic of Korea

**Keywords:** natural compounds, essential oil, antifungal agent, *Aspergillus flavus*, aflatoxin, mechanism of action

## Abstract

*Aspergillus flavus* is a facultative parasite that contaminates several important food crops at both the pre- and post-harvest stages. Moreover, it is an opportunistic animal and human pathogen that causes aspergillosis diseases. *A. flavus* also produces the polyketide-derived carcinogenic and mutagenic secondary metabolite aflatoxin, which negatively impacts global food security and threatens human and livestock health. Recently, plant-derived natural compounds and essential oils (EOs) have shown great potential in combatting *A. flavus* spoilage and aflatoxin contamination. In this review, the in situ antifungal and antiaflatoxigenic properties of EOs are discussed. The mechanisms through which EOs affect *A. flavus* growth and aflatoxin biosynthesis are then reviewed. Indeed, several involve physical, chemical, or biochemical changes to the cell wall, cell membrane, mitochondria, and related metabolic enzymes and genes. Finally, the future perspectives towards the application of plant-derived natural compounds and EOs in food protection and novel antifungal agent development are discussed. The present review highlights the great potential of plant-derived natural compounds and EOs to protect agricultural commodities and food items from *A. flavus* spoilage and aflatoxin contamination, along with reducing the threat of aspergillosis diseases.

## 1. Introduction

**Figure 1:**
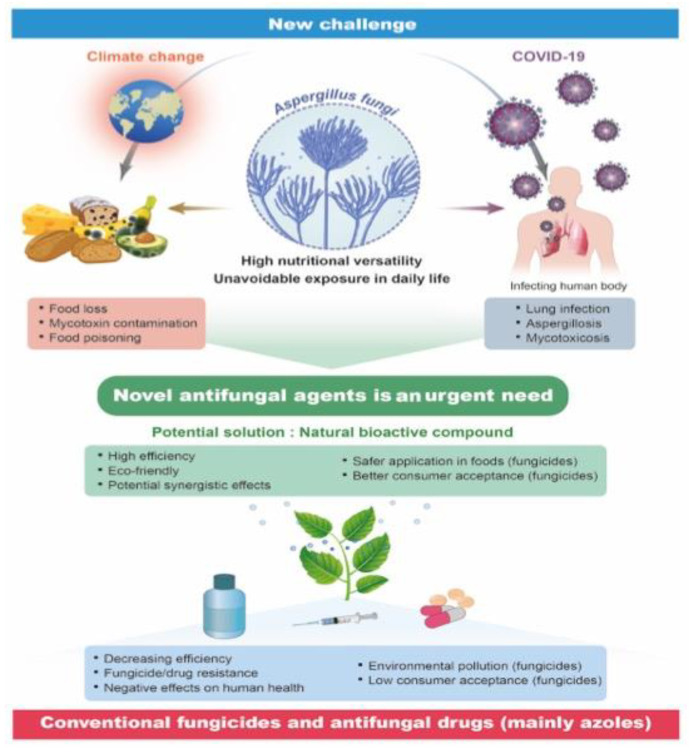
A schematic of the threat posed by *Aspergillus flavus* and the current measures available to combat it.

*Aspergillus flavus* is a facultative parasite that naturally exists as a saprophytic soil fungus and contaminates several important food crops at both the pre- and post-harvest stages. The fungus causes diseases in agricultural crops, such as cottonseed, maize, peanuts, and tree nuts, alongside being an opportunistic animal and human pathogen that causes aspergillosis diseases (Figure 1) [1]. *A. flavus* produces aflatoxin, which is a carcinogenic and mutagenic polyketide secondary metabolite. Aflatoxin contamination represents a worldwide food safety concern that impacts both the marketability and safety of multiple food crops [2]. The global economic losses due to their contamination have been estimated to be in the hundreds of millions of dollars, while maize and peanuts are the most affected food crops. For example, the US Food and Drug Administration (FDA) estimates that aflatoxin contamination in maize alone could cause annual losses to the food industry ranging from 52.1 million USD to 1.68 billion USD. Although most aflatoxigenic fungi commonly grow in tropical and subtropical climates (35° N–35° S), zones with a perennial aflatoxin contamination risk have expanded due to global climate changes. *A. flavus* and aflatoxin contaminations are predicted to pose serious threats to many countries and regions in the near future [3].

*A. flavus* also colonizes the soil and decays vegetation, which makes avoiding exposure to this fungus at home, at the workplace, or even during hospitalizations almost impossible [2]. Indeed, *A. flavus* is also isolated comparatively at a higher frequency from aspergillosis infections in humans, especially in developing countries. According to the US Centers for Disease Control and Prevention (CDC), the number of hospitalizations related to aspergillosis in the United States increased by an average of 3% per year during 2000–2013. Moreover, nearly 15,000 aspergillosis-associated hospitalizations occurred in the United States in 2014, at an estimated cost of 1.2 billion USD. Interestingly, most people breathe in *Aspergillus* spores every day without becoming ill. However, people with weakened immune systems or lung diseases are at a higher risk of developing health problems due to *Aspergillus*. For example, recent studies on ventilated patients with COVID-19 have reported a higher incidence of aspergillosis, affecting up to 30% of intubated patients [4].

The control of *A. flavus* and aflatoxin contamination usually focuses on inhibiting the development of spores and mycelia and/or the inactivation of aflatoxins by their transformation into nontoxic compounds. These processes depend primarily on chemical and physical approaches, including the use of synthetic fungicides, ozone fumigation, irradiation, dehulling or cooking processes, and manipulating environmental factors during harvests and storage. Most of the current strategies are expensive, time-consuming, and inefficient, while some even fail to protect the food without causing major changes in its physical properties and a serious loss of nutritive value. The continued application of synthetic fungicides is still the most effective and widely used recourse to control *A. flavus* and aflatoxin contamination. Synthetic fungicides, represented by azoles, which act by inhibiting the fungal cell membrane synthesis, are currently the major recourse for preventing *Aspergillus* contamination in food. However, despite the strict regulations on the application of chemical compounds in food, the use of synthetic fungicides can induce notable side effects, including toxicity to humans and animals, environmental pollution, and the development of drug-resistant fungal pathogens [5,6]. For example, triazole fungicides are one of the most widely used broad-spectrum fungicides. Human exposure to triazole fungicides induces various adverse health effects, including developmental and hepatic toxicities, liver carcinogenicity, and reproductive toxicities. Recent studies also suggest that many triazole fungicides are potential endocrine disruptors and could interfere with steroid hormone biosynthesis in mammals [7]. Strobilurin fungicides are another group of fungicides that have been widely used in agriculture for decades. Studies have confirmed the cytotoxicity and genotoxicity of strobilurin fungicides to human peripheral blood lymphocytes [8]. Aspergillosis treatments also depend on the use of azole antifungal drugs (e.g., voriconazole and itraconazole), to which an increasing number of fungal species have developed resistance. Meanwhile, consumers’ demand for healthier and more sustainable food products is also increasing. In response, the food industry is reformulating its products to replace artificial food additives and preservatives with natural, label-friendly alternatives [9]. Therefore, the development of novel antifungal agents that effectively inhibit the growth, mycotoxin biosynthesis, and pathogenicity of *A. flavus* is in urgent need.

Most plants produce natural antimicrobial agents, either as part of their normal growth and development or in response to microbial infections or environmental stresses. Many plant-derived natural compounds and herbal extracts, such as phenols, terpenes, and terpenoids, have great potential for utilization in the combat of both foodborne and human pathogens [9]. Besides being highly effective, many plant-derived natural compounds are safe for human health and biodegradable, which makes them attractive substitutes for synthetic preservatives. Although plant-derived natural compounds seem promising for the development of novel antifungal and antiaflatoxigenic strategies against *A. flavus*, the lack of in situ and in vivo studies regarding their activities and the limited understanding of their mechanisms of action, as well as the lack of cost-benefit and ecotoxicity assessments, have largely deterred their application in the development of novel antifungal agents. Furthermore, standardized commercial productions require the consideration of multiple factors, including plant varieties, plant nutrition, geographic location, seasonal variation, surrounding climate, agronomic practices, and methods of extraction and storage [10]. Therefore, further studies are required to understand their physicochemical properties and possible mechanisms of action. Hence, this review aims to provide an overview of the published data on these issues. Moreover, the information provided here can facilitate the implementation of plant-derived natural compounds as natural antifungal agents in food preservation and human health protection.

## 2. Antifungal and Antiaflatoxigenic Activities of Plant-Derived Natural Compounds against *A. flavus*

Plant-derived natural compounds are known to have broad-spectrum antimicrobial activities and diverse effects on microbial physiological and metabolic activities. Many studies have demonstrated that plant-derived natural compounds or essential oils (EOs) exhibit antifungal and antiaflatoxigenic activities against *A. flavus*. Many previous studies were conducted in vitro using direct-contact antimicrobial assays [10,11,12,13,14,15]. Some plant-derived natural compounds have been similarly tested on food materials, such as corn, wheat, soybean, chickpea, pistachio, peanut, and rice (Table 1). The most widely invested plant-derived natural compounds or EOs against *A. flavus* and aflatoxin contamination in food materials are isolated from food-flavoring plants, such as clove (*Syzygium aromaticum* L.), cinnamon (*Cinnamomum zeglanicum*, *C. verum*), oregano (*Origanum vulgare* L.), and thyme (*Thymus vulgaris* L.). These plants and their EOs or extracts have been used in food preparation for centuries and are categorized as “generally recognized as safe” (GRAS) by the U.S. FDA. Moreover, many of them possess beneficial effects on the human body, which, combined, makes them the ideal sources of safe, natural, antifungal, and antiaflatoxigenic agents.

The antifungal agents extracted from different plants are highly diverse [54,55]. Most of the plant-derived natural compounds that exhibit antifungal activity are phenols, terpenes, and terpenoids [9]. The antimicrobial activity of phenols is closely connected with their hydroxyl group, which is bonded directly to a benzene ring. The presence of a free hydroxyl group enables phenols to exchange their proton, thus, promoting their ability to modify the cell membrane integrity of microbes [56]. Meanwhile, the hydroxyl group’s relative position on the benzene ring can potentially affect their antimicrobial efficacy. Tian et al. investigated the structure–activity relationships of plant-derived flavonoids against *A. flavus* growth and aflatoxin biosynthesis. Flavonoids represent the largest group of naturally occurring phenolic compounds, and Tian et al. identified that the [–OH] or [–O–CH_3_] groups at position 6 of ring A and position 4′ of ring B are closely associated with the antifungal and antiaflatoxigenic activities of natural flavonoids [57]. Regarding terpenes, the connection between their structure and functional groups and their antimicrobial activities remains unclear. Previously, the number of double bonds and acyclic, monocyclic, and bicyclic structures in terpenes has been shown to limit their antimicrobial activities [9,58]. Terpenoids are suggested to possess greater antimicrobial activities than most terpenes, and these activities are mainly determined by their functional groups, such as alcohols and aldehydes [59]. Furthermore, their hydrogen-bonding capacity and relative solubility could potentially affect their antimicrobial activities.

The efficiency of plant-derived natural compounds against fungal growth and mycotoxin production is affected by environmental factors, including light, oxygen, pH, temperature, and water activity. Light exposure and high amounts of oxygen in packaging generally decrease the antimicrobial efficiency of plant-derived natural compounds, probably through oxidation [60]. Passone et al. reported that exposing boldo (Pëumus boldus Mol.) to UV light and sunlight for 30 min reduced the EOs’ antifungal activity by 29.1% and 29.6%, respectively [61]. The antimicrobial effects of plant-derived natural compounds tend to increase as environmental pH levels decrease [62]. The hydrophobicity of plant-derived natural compounds has been reported to increase at low pH levels since these dissolve in the lipids of microbial cell membranes more easily. Temperature is a key factor in the growth and mycotoxin production of *A. flavus*. The susceptibility of microbials to plant-derived natural compounds generally increases as storage temperatures decrease [60]. Meanwhile, the antifungal ability of plant-derived natural compounds is usually stable against alterations in temperature. Passone et al. evaluated the antifungal activity of EOs in boldo (*P. boldus* Mol.) and poleo (*Lippia turbinata*) following exposure to various temperatures (40, 60, and 80 °C). They observed the same level of inhibition against *A. flavus* [61]. Sharma and Tripathi tested the thermostability of *Citrus sinensis* L. EOs against *A. niger* following exposure to temperatures of 40, 60, 80, 100, and 121 °C and found that the activity of *C. sinensis* EOs did not change after any of the treatments [62]. Furthermore, the antifungal and antiaflatoxigenic efficiencies of EOs are closely dependent on the water activity of the substrate. Generally, as the water activity decreases, the fungal growth is unfavored, and the antifungal activity of the EOs is promoted. The antiaflatoxigenic effects of EOs increase at relatively low water activities [61].

The interactions between individual plant-derived natural compounds may cause synergistic or antagonistic effects. For example, several studies report that a mixture of the major components of an essential oil exhibits weaker antibacterial activity than the whole EO, most likely due to the remaining missing components [63,64]. Currently, the majority of studies regarding the synergistic antifungal effects of plant-derived natural compounds have focused on the interactions of phenols, terpenes, and terpenoids. Songsamoe et al. tested the synergistic effect of linalool and caryophyllene and found that a 10:1 ratio was key to enhancing the antifungal activity of M. alba EOs against *A. flavus* [44]. Stević et al. likewise demonstrated that the synergistic activity between thymol and carvacrol plays an important role in the overall antifungal activity of thyme and oregano EOs [65]. The synergistic antifungal effects of different EOs have been further reported. For example, a combination of oregano and thyme EOs resulted in a synergistic antifungal effect against a variety of food pathogens, including *A. flavus*, *A. parasiticus*, and *P. chrysogenum*. Moreover, mixtures of peppermint and tea tree EOs exhibited synergistic effects against *A. niger* [66]. However, it remains extremely difficult to predict the antimicrobial efficacy of EO mixtures since each EO is a complex mixture of different chemical compounds, and interactions between the individual components can produce both synergistic and antagonistic antimicrobial effects [67]. The interaction between EO compounds and those in the food matrix could potentially affect the antifungal and antiaflatoxigenic efficiencies of EOs. Tian et al. evaluated the antiaflatoxigenic activity of thyme EOs in brown and white rice [48]. Here, thyme EOs reduced aflatoxin accumulation by up to 72.7% in brown rice but by only 18.0% in white rice. Brown rice contains higher levels of fiber, minerals, phenolic compounds, protein, and vitamins compared with white rice [68], which may partly contribute to its resistance to aflatoxin contamination. Further suggestions include a potential interaction between the EO’s phenolic compounds and those in white rice, while the possible synergistic effects between these bioactive constituents can promote the antiaflatoxigenic activity of this EO.

The synergistic antifungal effects of plant-derived natural compounds with fungicides and antifungal drugs have also been reported. For example, baicalein, a flavonoid originally isolated from *Scutellaria baicalensis* and *Scutellaria lateriflora*, showed no antifungal effects against *A. flavus* when tested at a concentration of 4 μg/mL in an in vitro test. However, at the same concentration, baicalein was able to reduce the 80% effective concentrations of strobilurins (azoxystrobin and pyraclostrobin) from 4 μg/mL and 0.125 μg/mL to 0.5 μg/mL and 0.016 μg/mL, respectively [69,70]. Cinnamaldehyde was reported to show strong synergy with fluconazole antifungal drugs against the human pathogenic fungus *A. fumigatus* by reducing the minimum inhibitory concentration of fluconazole from 200 μg/mL to 25 μg/mL in vitro [71]. Plant-derived natural compounds are suggested to promote the antifungal activity of fungicides and drugs by increasing the bioavailability of co-administered drugs in fungal cells through the inhibition of metabolizing enzymes, such as cytochrome P450 isoforms and multi-drug or efflux transporters [72,73,74]. Plant-derived natural compounds are able to boost the antifungal efficiency of commonly used antifungal agents while reducing their required amounts for the effective inhibition of fungal contamination and infections, and, hence, they may lower the potential toxic effects on humans. Therefore, plant-derived natural compounds represent a good source of combined agents for antifungal treatments or therapies. Further studies on the in vivo and in situ synergistic effects of plant-derived natural compounds with fungicides and antifungal drugs are of great significance.

## 3. Antifungal Mechanisms of Plant-Derived Natural Compounds and EOs against *A. flavus*

### 3.1. Acting on the Cell Wall of A. flavus

**Figure 2:**
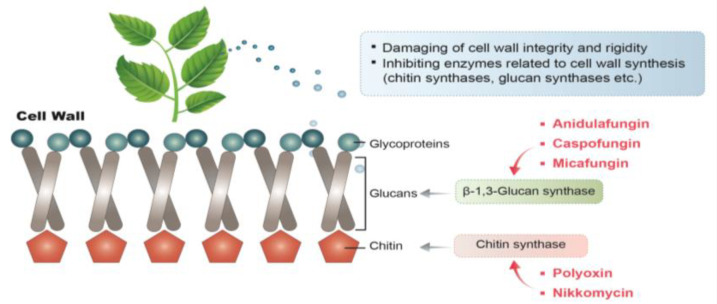
Mechanisms of action of plant-derived natural compounds and antifungal agents acting on the cell wall of *A. flavus*.

Different plant-derived natural compounds may target various kinds of microbes and execute their antimicrobial activity through numerous mechanisms. Meanwhile, the antifungal effects of natural compounds often have multiple targets, including the cell wall, cell membrane, mitochondria, and metabolic enzymes. Accordingly, their antifungal mechanisms of action may be due to physical, chemical, or biochemical changes in these cell components. Fungi have a unique cell wall structure that is absent in humans. It is comprised of a complex and dynamic structure of chitin, glycans, and glycoproteins, which fulfills several essential functions connected with the interaction between the cell and its environment. Indeed, the fungal cell wall is involved in the morphogenesis of fungal cells, the protection of the protoplasts from physical damage and osmotic stress, cell recognition in various interactions, and the exchange of nutrients and metabolic products, alongside drug resistance against antifungal agents [75]. Cumulatively, these properties make the fungal cell wall a promising target for antifungal agents. Chitin and glucan are mainly located in the inner layer of the fungal cell wall. In most filamentous fungi, chitin consists of β-1,4-linked N-acetylglucosamine and is covalently linked to β-1,3-glucan, which provides the structural integrity and physical strength of the cell wall [28,29]. Therefore, the disruption of chitin biosynthesis causes disorder in the fungal cell wall, which leads to cell lysis and death [26]. Many successful antifungal agents, such as nikkomycin and polyoxin, perform their antifungal activity by specifically inhibiting fungal chitin synthase (Figure 2). Another major cell wall polysaccharide in filamentous fungi is β-1,3-glucan, which is essential for proper cell wall formation and normal cellular development [27]. Antifungal agents, such as caspofungin, micafungin, and anidulafungin, inhibit β-1,3-glucan synthase and, consequently, can disrupt the fungal cell wall formation, preventing fungal growth. In the outer layer of the fungal cell wall, cell wall-associated proteins are covalently bound to mannan and form mannosylated glycoproteins [34,35,36]. These proteins perform a wide range of functions and are involved in various cellular activities, including cell-to-cell interactions, trafficking of nutrition and macromolecules, providing protection against toxic substances, and cell surface adhesion.

The antifungal activity of numerous plant-derived natural compounds and EOs was reported to damage the integrity and rigidity of the *A. flavus* cell wall. For example, paeonol (2-hydroxy-4-methoxyacetone), which is an active component commonly isolated from *Cynanchum paniculatum*, is reported to alter the cell wall ultrastructure and reduce the content of both β-1,3-glucan and chitin in *A. flavus* [76]. Similarly, extracts of *Tulbaghia violacea* were found to reduce the content of β-1,3-glucan and chitin in the *A. flavus* cell wall [77]. Indeed, da Silva and co-workers tested the antifungal effects of *R. officinalis* L. EOs and observed significant microscopic morphological changes, including cell wall rupturing and cytoplasmic leakage [78]. Such modifications are potentially associated with plant-derived natural compounds interfering with the enzymes related to cell wall synthesis. The building blocks of the fungal cell wall, such as chitins, glucans, and pectins, are continuously remodeled to cope with fungal growth and development by different enzymes, such as chitin and glucan synthases, glycohydrolases, and transglycosidases [79]. Fungicides have been developed by targeting the related enzymes, including polyoxins (which inhibit chitin synthesis), echinocandins (which inhibit β-1,3-glucan synthase), and tricyclazole (which inhibits melanin synthesis). Several plant-based natural compounds are known to inhibit cell wall synthases. Indeed, Marei et al. found that thymol and limonene had inhibitory effects on fungal cellulase and pectin methyl esterase [80]. Additionally, Bang et al. reported that cinnamaldehyde inhibits chitin synthase and β-1,3-glucan synthase [81].

### 3.2. Acting on the Cell Membrane of A. flavus

**Figure 3:**
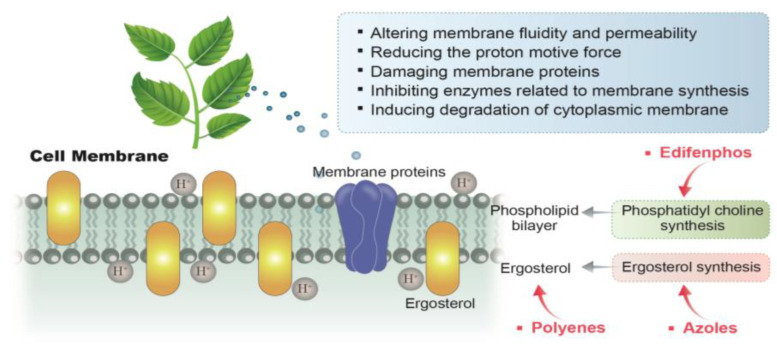
Mechanisms of action of plant-derived natural compounds and antifungal agents acting on the cell membrane of *A. flavus*.

Fungal membranes have been proposed as the major targets for the antifungal activity of plant-based natural compounds (Figure 3). The ability to pass through the cell wall and penetrate the cell membrane is fundamental for their antifungal activity. Natural compounds may affect fungal membranes through several mechanisms of action, including: (1) altering membrane fluidity and permeability; (2) reducing the proton motive force; (3) damaging membrane proteins; (4) inhibiting enzymes related to membrane synthesis; (5) inducing cytoplasmic membrane degradation [82,83,84]. These alterations in the cell membrane result in the loss of cell homeostasis, leakage of cell components, and, ultimately, cell death. Plant-based natural compounds, such as thymol, carvacrol, and eugenol, have been reported to cause ion imbalance across the cell membrane by dissipating H+ and K+ ion gradients, thus, facilitating the leakage of vital cellular components and inducing water stress, alongside intracellular ATP depletion [85]. Numerous plant-based natural compounds act by binding to membrane ergosterol or inhibiting its biosynthesis. Ergosterol is the main sterol derivative of fungi and is essential for preserving cell membrane functionality and maintaining the cell’s integrity, viability, and normal growth functions [86]. Since ergosterol is essential to fungal cells, antifungal drugs and fungicides have been developed and widely applied to act through the same mechanism in both clinical and agricultural antifungal practices. Among them, polyenes (which bind to ergosterol) and azoles (which inhibit ergosterol synthesis) are the most successful. Freires and co-workers found that coriander (*C. sativum* L.) EO could bind to the fungal membrane ergosterol and increase ionic permeability to cause membrane damage, leading to cell death [87]. Tian et al. reported that *C. jensenianum* EOs inhibited ergosterol biosynthesis in *A. flavus* [88]. A similar effect was also detected with natural compounds extracted from dill (*A. graveolens* L.), thyme (*T. vulgaris* L.), and turmeric (*C. longa* L.) [49,89,90]. Moreover, treatments with the plant-derived natural monoterpene citral downregulated ergosterol biosynthetic genes (e.g., *erg7*, *erg11*, *erg6*, *erg3*, and *erg5*) [91]. Lipophilic natural compounds, such as phenols and aldehydes, can pass through the double phospholipid bilayer and interact with ergosterol or enter the nucleus and regulate its biosynthetic genes. Ultimately, this causes cell membrane modification, fatty acid profile alterations, and osmotic imbalances, which lead to irreversible damage to the membrane and morphological alteration in hyphae, conidiophores, and conidia and, finally, cell death [9]. The attachment or penetration of the natural compounds via the fungal membranes may prompt the cell structures to disintegrate, causing the fungal cells to become more permeable to the compounds. Phenols, such as thymol, carvacrol, and eugenol, possess a system of delocalized electrons and may also reduce the pH gradient across the cytoplasmic membrane by acting as proton exchangers. The collapse of the proton motive force and the depletion of the ATP pool, resulting from such an effect, can lead to the leakage of iron and intracellular cell constituents and, eventually, cause cell death [92]. Terpenoids also execute their antimicrobial activity at the cell membrane level. It has been reported that terpenoids disrupt the membrane permeability by altering the membrane fatty acid composition and ergosterol content, which results in leakage of the cell contents [93].

### 3.3. Acting on the Mitochondria of A. flavus

**Figure 4:**
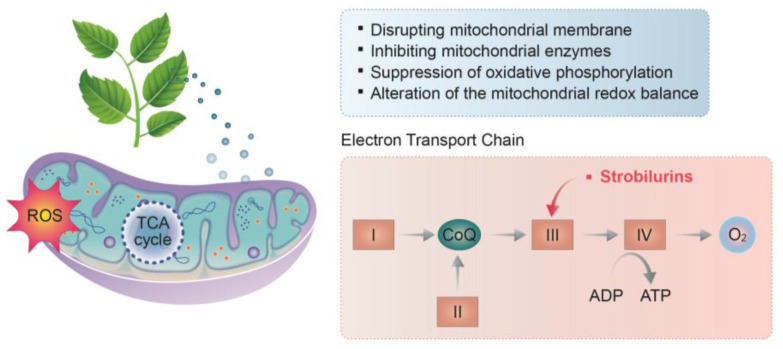
Mechanism of action of plant-derived natural compounds and antifungal agents acting on the mitochondria of *A. flavus*. I, II, III, IV, mitochondrial complex I, II, III, IV.

Mitochondria play wide-ranging roles in fungal cells. Mostly known for their role in cellular respiration, mitochondria are also associated with other important cellular functions related to virulence, developmental and morphogenetic transitions, drug resistance, ergosterol biosynthesis, and cell wall maintenance [94]. The respiratory chain has been proven to be an effective target for fungicides to control fungal contamination in food crops. Quinone outside-inhibiting (QoI) fungicides, represented by strobilurins, are the most important group of fungicides developed for respiration inhibition (Figure 4). QoI fungicides inhibit fungal pathogens by blocking the transfer of electrons at the quinone outer binding site of the mitochondrial complex III, thus, reducing the production of ATP, which leads to a reduction in the normal metabolic functions and, eventually, cell death. Plant-derived natural compounds and EOs also appear to inhibit fungal growth by damaging mitochondria. For example, (E)-2-hexenal, a leaf volatile produced naturally by green plants as a defense response, was found to inhibit the mitochondrial dehydrogenases and disrupt the mitochondrial energy metabolism of *A. flavus* [95,96]. Honokiol, a phenolic compound isolated from the plant *Magnolia officinalis*, caused mitochondrial hyperpolarization and dysfunction and led to ATP depletion in *A. flavus* [97]. Dill (A. graveolens L.) EOs were reported to reduce *A. flavus* growth by promoting mitochondrial dysfunction and the accumulation of reactive oxygen species (ROS) [90]. Tea tree EO was also found to damage the mitochondria of *Botrytis cinerea* [98]. However, the exact mechanisms through which dill and tea tree EOs function are not fully understood. It has been suggested that these EOs may contain natural compounds that can modulate mitochondrial functions by (1) disrupting the mitochondrial membrane, (2) inhibiting mitochondrial enzymes, (3) suppressing oxidative phosphorylation, and (4) altering the mitochondrial redox balance. Li et al. reported that tee tree EOs can destroy the mitochondrial morphology and function in *B. cinerea* by increasing the mitochondrial membrane permeability and decreasing the enzymatic activities related to the tricarboxylic acid (TCA) cycle (malic dehydrogenase, succinate dehydrogenase, ATPase, citrate synthetase, isocitrate dehydrogenase, and α-ketoglutarate dehydrogenase). These alterations subsequently result in matrix loss, accumulation of ROS, and increased mitochondrial irregularity [98]. Zheng et al. determined that citral exerted antifungal activity by altering the mitochondrial morphology, which resulted in the loss of the mitochondrial matrix and mitochondrial collapse [99]. Tian et al. also detected mitochondrial morphological alterations in *A. flavus* treated with dill EOs and suggested that these are caused by an increase in the mitochondrial membrane permeability, malfunctions in the cellular metabolism, and a breakdown of the mitochondrial matrix [90]. Additionally, plant-based natural compounds may be able to damage fungal mitochondria by disrupting the osmotic balance, causing calcium and protons to leak, and, consequently, altering the electrochemical potentials. For example, Rao and co-workers reported that carvacrol was able to induce calcium stress in yeast’s mitochondria through the activation of the target of rapamycin (TOR) signaling pathway [100]. Some EOs contain natural compounds that can induce mitochondrial dysfunction via the inhibition of mitochondrial enzymes. For example, turmeric (*C. longa* L.) EOs were found to inhibit ATPase, malate dehydrogenase, and succinate dehydrogenase in *A. flavus* mitochondria, thus, suppressing its contamination in maize [49]. Dill EOs were also found to inhibit fungal ATPase [101]. Phenols and aldehydes in plants have been suggested to be the major natural compounds that interact with mitochondrial enzymes [102].

## 4. Antiaflatoxigenic Mechanisms of Plant-Derived Natural Compounds and EOs against *A. flavus*

Aflatoxin is a polyketide-derived furanocoumarin; a type of carcinogenic and mutagenic secondary metabolite that threatens global food security. Many studies suggest that reduced fungal growth is not solely responsible for the inhibition of aflatoxin production by plant-based natural compounds. Instead, the specific inhibition of aflatoxin biosynthesis is also thought to play a role. Different plant-derived natural compounds have been proven to inhibit the production of aflatoxin at lower concentrations than those that inhibit mycelial growth. For example, Tian et al. tested the antiaflatoxigenic activity of 36 structurally related natural flavonoids isolated from plants. Baicalein, flavone, hispidulin, kaempferol, and liquiritigenin were found to exclusively exhibit significant antiaflatoxigenic activity (>80% inhibition compared to the control), despite having none or very low antifungal activity [57]. Aflatoxin production inhibitors that do not affect fungal growth may also be useful as selective aflatoxin control agents without significantly interrupting the microbial environment or incurring the rapid spread of resistant strains.

**Figure 5:**
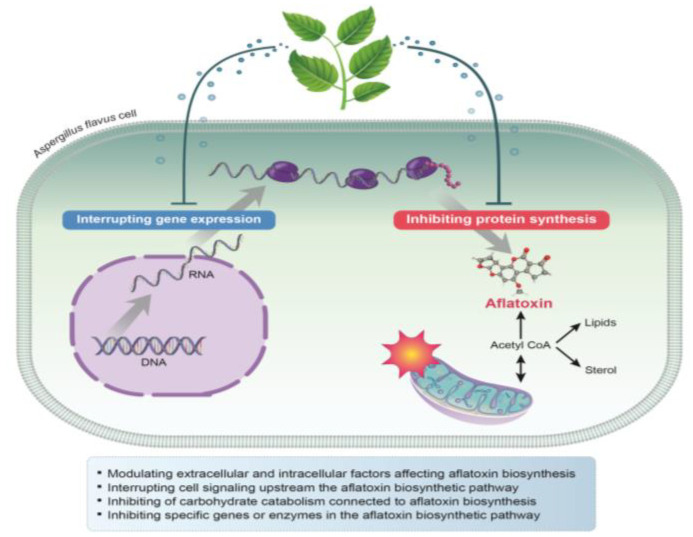
Mechanisms of action of plant-derived natural compounds against aflatoxin biosynthesis of *A. flavus*.

Currently, the antiaflatoxigenic modes of action of plant-based natural compounds are not clearly understood. However, over the last few decades, it has been hypothesized that natural compounds may inhibit aflatoxin biosynthesis through: (1) modulating extracellular and intracellular factors affecting aflatoxin biosynthesis; (2) interrupting the cell signaling upstream of the aflatoxin biosynthetic pathway; (3) inhibiting carbohydrate catabolism, which is connected to aflatoxin biosynthesis; (4) inhibiting specific genes or enzymes in the aflatoxin biosynthetic pathway (Figure 5) [103]. In addition, the antiaflatoxigenic activity of natural compounds is potentially related to the transduction and perception of signals involved in the switch of fungal cells from vegetative to reproductive development. The correlation between secondary metabolism and fungal sporulation has been highlighted in many studies [22,104].

In particular, the mitochondria in *A. flavus* also play critical roles in the biosynthesis of aflatoxin. Mitochondria are responsible for providing ATP, NADPH, and acetyl-CoA for aflatoxin biosynthesis. Plant-based natural components may disrupt the mitochondria and cease the formation of acetyl-CoA that feeds the aflatoxin biosynthesis pathway, ultimately leading to the inhibition of aflatoxin biosynthesis. Mitochondria are also involved in many important fungal cellular activities that affect aflatoxin production, such as regulating fungal development, directing cellular metabolism, and maintaining cellular ROS levels. Many secondary fungal metabolites, including aflatoxin, are synthesized when the fungus finishes its initial growth phase and begins its development stage, which is represented by sporulation [22,104]. Moreover, mitochondria have been suggested to contribute to the regulation of both the physiological and morphological developments of fungal cells [105]. Mitochondria also regulate the lipid metabolism in fungal cells. It has been suggested that on initiation of aflatoxin biosynthesis, the external carbon source was greatly consumed, thus, resulting in aflatoxin production from the breakdown of reserve carbon sources, mostly lipids and fatty acids [106]. Additionally, some natural compounds’ antiaflatoxigenic activities may be related to the inhibition of lipid peroxidation and oxygenation, which are processes involved in aflatoxin biosynthesis [107]. Studies on *A. flavus* have revealed the importance of oxylipins in the regulation of aflatoxin biosynthesis, conidia production, and sclerotia formation [108]. Mitochondria may also affect aflatoxin synthesis via a ROS-related mechanism. Studies have shown that aflatoxin biosynthesis involves a boost in the oxygen uptake of fungal cells, followed by an increase in ROS generation. This change occurs when fungal cells switch from trophophase to idiophase, at which point different secondary metabolic pathways become active [109]. The stimulation of aflatoxin biosynthesis by ROS-induced oxidative stress has also been previously reported [110,111]. The presence of multiple cytochrome p450 monooxygenases and monooxygenases in the aflatoxin biosynthesis pathway also indicates the involvement of both oxygen consumption and ROS production in this system. Many natural compounds possess strong antioxidant activities that can scavenge the ROS inside the fungal cells. The antiaflatoxigenic mechanism of natural compounds may be associated with their antioxidant activity and ROS scavenging ability, which attenuates the fungal oxidative stress responses and reduces aflatoxin production [112].

Additional reports have also suggested that the antiaflatoxigenic activity of some natural compounds occurs via the inhibition of the carbohydrate metabolism by inhibiting some key enzymes [27]. Alternatively, it could occur by inhibiting the aflatoxin biosynthetic pathway by suppressing the expression of the aflatoxin biosynthetic genes. Indeed, the expression of internal transcriptional regulators (*aflR* and *aflS*) in the aflatoxin biosynthetic pathway is inhibited by natural phenols and terpenoids [113,114]. Furthermore, the expression of structural genes, such as *aflD*, *aflK*, *aflE*, *aflM*, *aflO*, *aflP*, *aflO*, and *aflQ*, is reported to be suppressed by different plant-derived natural components [49,113,114,115]. However, the mechanisms through which they exert their actions at the molecular level still need to be deciphered fully.

## 5. Future Perspective

**Figure 6:**
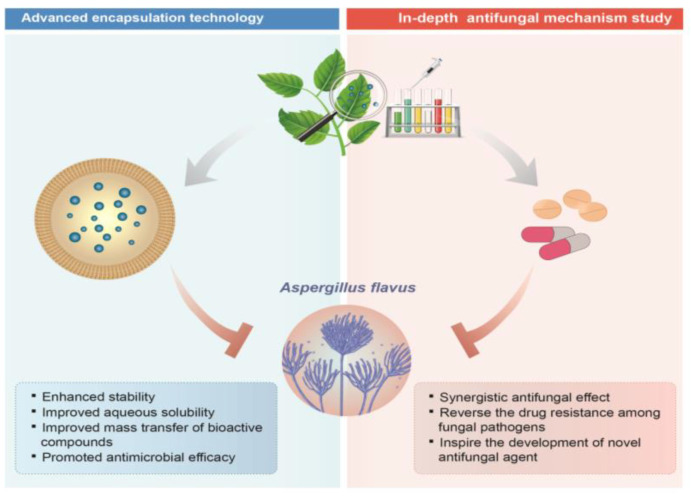
Future perspectives towards the application of plant-derived natural compounds against *A. flavus*.

Recently, consumers’ demands for healthier and more sustainable food products have required the food industry to reformulate their products by replacing artificial additives with natural, label-friendly alternatives [9]. Many plant-derived natural compounds and EOs have exhibited great potential against fungal propagation and mycotoxin production. Many present either no mammalian toxicity or a lower one than synthetic fungicides, which had a higher lethal dose of 50 values during toxicological testing using oral administration in mice [116,117,118]. Presently, natural compound-based fungicides are being developed and are available in European countries and the USA, e.g., Fungastop^TM^ and Armorex^TM^ II (Soil Technologies Corp., Fairfield, IA, USA). Future studies may focus on improving the stability and performance of plant-derived natural antifungal compounds and EOs to allow for their application in foods and complex biological systems, in addition to an in-depth investigation into their antifungal mechanisms (Figure 6).

The extraction process for plant-derived bioactive compounds and EOs plays an important role in both their yield and quality. Considering their low amounts in plants, it is desirable to use methodologies that can result in maximum yields with a minimum loss in functional properties. Plant-derived bioactive compounds encompass a wide range of natural metabolites with different physiochemical qualities. The extraction of these compounds is strongly dependent on the hydrophobic or lipophilic characters of the target molecules. Currently, various innovative extraction methods have been proposed for the extraction of bioactive compounds and EOs from plant materials, including high pressure extraction, high voltage electrical discharges, microwave-assisted extraction, pressurized liquid extraction, pulsed electric fields-assisted extraction, sub- and supercritical fluid extraction, and ultrasound-assisted extraction [119,120,121]. These techniques are able to reduce the usage of chemical solvents and energy during the extraction process and improve the yields and quality of EOs.

Despite the great interest in the exploitation of natural compound-based food preservatives, their large-scale utilization is currently limited, as are their antifungal and antimycotoxigenic activities [122]. The major limitations regarding the application of plant-derived natural compounds in food commodities include low water solubility, instability, susceptibility to oxidation, volatilization, and rapid degradation in different environmental conditions [123]. Meanwhile, due to the low stability of many natural components, their antifungal and antimycotoxigenic effects tend to be compromised following long-term storage. One way to mitigate this effect is to develop suitable structural barriers to enclose the natural compounds. In this regard, modern encapsulation techniques that use different physical, physicochemical, and mechanical methods with the assistance of carrier matrices have been tested for the formulation of natural compounds or EO-based preservatives. The encapsulation of plant-derived natural compounds and EOs can reduce the loss of bioactivity and offer the possibility of controlling the release in treated foods [124]. Thus, emulsion-based delivery systems and active packaging materials, formulated with the incorporation of natural compounds, have been tested to improve the aqueous solubility and mass transfer of natural antimicrobial compounds in foods. In particular, nanotechnology, as a quickly growing field, has been applied to a variety of marketable products around the world. Nanoencapsulation of bioactive compounds and nanoemulsified EOs have demonstrated the advantages of more efficient and targeted use of antifungal agents in a safer and environmentally friendly way [125,126,127,128]. Such advantages were associated with their distinct physicochemical and functional characteristics and enhanced ability to transport bioactive constituents through biological membranes. Therefore, nanotechnology has the potential to significantly improve the antifungal efficiency of plant-derived natural compounds and EOs in practical applications.

Currently, the primarily used antifungal drugs and fungicides exert their antifungal activities by targeting the fungal cell wall, cell membrane, mitochondria, or important metabolic enzymes. Plant-derived natural antifungal compounds that act on either the same or connected cellular structures can potentially enhance the antifungal effects of the fungicides. Natural compounds that disrupt cell membrane functions or ATP production may also inhibit the activity of drug efflux pumps, thus, decreasing the drug resistance of fungal cells. Natural compounds that interact with the cellular redox system may improve the antifungal effects of mitochondria-targeting antifungal agents [129]. In these cases, the natural compounds function by attenuating the ability of the fungal cells to activate the defense response against fungicides. Hence, these do not necessarily require a great degree of antifungal potency to be effective. The application of these natural compounds, in combination with conventional fungicides, can lower the dosage levels required to control the pathogens and reduce the costs and risks of negative side effects. Such natural compounds could make the use of conventional fungicides safer and more effective, while also overcoming the development of drug resistance in both food and human fungal pathogens.

## Figures and Tables

**Table 1:** In situ antifungal and antiaflatoxigenic activities of plant-derived natural compounds against *A. flavus* in different food systems.

Name of the Plant	Major Components	Food Systems	Antifungal Activity	Antiaflatoxigenic Activity	References
*Ageratum conyzoides*	β-Caryophyllene; germacrene-D; dimetoxy ageratocromene	Wheat; corn; soybean	80.8% inhibition at 1 μL/mL (vapor) in wheat grain (12 months); 79.5% and 100% inhibition in corn and soybeans, respectively, with 5 μL EO (disk diffusion assay, 5 days)	93.7% reduction with 50 μL in 60 g corn (direct contact) (10 days); >75% reduction with 50 μL in 60 g in soybeans (direct contact) (10 days)	[16,17]
Ajowan (*Trachyspermum ammi* L.)	*p*-Cymene; thymol	Wheat; chickpea	46.2% and 65.2% inhibition at 0.8 μL/mL (vapor) in wheat and chickpea, respectively (12 months)	100% inhibition at 0.8 μL/mL (vapor) in wheat and chickpea (12 months)	[18]
Anise (*Pimpinella anisum* L.)	Anethol	Corn; wheat	MIC: 1000–3000 μg/g (dependent on aw) in corn (11 days); 100% inhibition at 1% (*v*/*w*) in wheat (14 days)	100% inhibition at 1000 → 3000 μg/g (dependent on aw) in corn (11 days); 100% inhibition at 1% (*v*/*w*) in wheat (8 weeks)	[6,19]
Boldo (*Pëumus boldus* Mol)	α-Terpinolene; α-terperpine; *p*-cimene	Corn	MIC: 500–2000 μg/g (dependent on aw) (11 days)	100% inhibition at 500–2000 μg/g (dependent on aw) (11 days)	[6]
*Boswellia carterii* Birdw	Phenylethyl alcohol; benzyl acetate	Pepper fruits (*Piper nigrum* L.)	65.4% inhibition at 1.75 μL/mL (vapor) (6 months)	No record	[20]
*Boswellia serrata*	3-Carene; β-ocimene	Corn	No record	95.6% inhibition at 10 μL/g (10 days)	[21]
*Cananga odorata*	β-Caryophyllene	Chickpea	77.4% inhibition at 2 μL/mL (vapor) (6 months)	No record	[22]
Caraway (*Carum carvi*)	Limonene; carvone	Bread; polenta	90% inhibition at 636.47 μL/L (vapor) in bread (14 days)	100% inhibition in bread requires > 500 μL/L of EO (vapor) (14 days); 100% inhibition at 4.5 μg/g in polenta (14 days)	[23,24]
*Carum copticum*	*p*-Cymene; γ-terpinene; thymol	Cherry tomato	58.0% inhibition at 100 μL/mL (vapor) (30 days)	No record	[25]
*Chenopodium ambrosioides* Linn.	α-Terpinene; *p*-cymene; Ascaridole	Pigeon pea	100% inhibition at 0.29 μL/mL (vapor) (6 months)	No record	[26]
*Cicuta virosa* L. var. *latisecta* Celak	*p*-Cymene; γ-terpinene; cuminaldehyde	Cherry tomato	89.9% inhibition at 200 μg/mL (vapor) (9 days)	No record	[27]
*Cinnamomum glaucescens*	1,8-Cineole; 2-propenoic acid	Chickpea	71.1% inhibition at 4.5 μL/mL (vapor) (12 months)	No record	[28]
Cinnamon (*Cinnamomum verum*)	Cinnamaldehyde; eugenol	Maize extract medium; bread	90% inhibition at 820 → 1000 mg/L (depending on temperature and water activity) in maize extract medium (12 days); 90% inhibition at 558.44 μL/L (vapor) in bread (14 days)	500–1000 mg/L (depending on temperature and water activity) in maize extract medium (12 days); 100% inhibition at 500 μL/L (vapor) in bread (14 days)	[23,29]
Cinnamon (*Cinnamomum zeglanicum*)	Cinnamaldehyde; eugenol; phenol, 2-methoxy-4-(2-propenyl)phenol	Pistachio; finger millet (*Eleusine coracana*); wheat	60–83% inhibition at 0.5 μL/mL (vapor) in finger millet (up to 6 months); 100% inhibition at 2% (*v*/*w*) in wheat (14 days)	100% inhibition with 25 mL of 9% (vol/vol) EO solution in 500 g pistachio (3 months); 100% inhibition at 2% (*v*/*w*) in wheat (8 weeks)	[19,30,31]
*Citrus reticulata*	Limonene; geranial; neral	Tuberous roots of *Asparagus racemosus*	68.6% inhibition at 500 ppm (*v*/*v*) (12 months)	61.76% inhibition at 500 ppm (*v*/*v*) (12 months)	[32]
*Clausena pentaphylla*	Sabinene; α-terpinolene; methyl eugenol	Pigeon pea seed	100% inhibition at 0.29 μL/mL (vapor) (6 months)	No record	[33]
Clove (*Caryophyllus aromaticus*)	eugenol	Pistachio	No record	100% inhibition with 25 mL of 9% (vol/vol) EO in 500 g pistachio (3 months).	[30]
Clove (*Syzygium aromaticum* L.)	Benzenemethanol; eugenol; eugeyl acetate; β-caryophyllene	Corn; Iranian white cheese; bread; tomato paste	100% inhibition at 500–3000 μg/g (dependent on water activity) in corn (11 days); 100% inhibition at 10 μL/L (vapor) in corn (5 days); 100% inhibition at 150 ppm in Iranian white cheese (up to 40 days); 90% inhibition at 674.49 μL/L (vapor) in bread (14 days); 48% inhibition at 500 ppm in tomato paste (2 months)	100% inhibition at 1000–2000 μg/g (dependent on water activity) in corn (11 days); 100% inhibition at 10 μL/L (vapor) in corn (5 days); 100% inhibition at 50–150 ppm in Iranian white cheese (up to 40 days); 100% inhibition at 500 μL/L (vapor) in bread (14 days);	[6,23,34,35,36]
*Coleus aromaticus*	Thymol; γ-terpinene; *p*-cymene	Wheat	87.37% inhibition at 0.1 μL/mL (vapor) (12 months);	No record	[16]
*Commiphora myrrha*	α–Elemene; curzerene; furanoeudesma-1,3-diene	Chickpea	55.4% inhibition at 3 μL/mL (vapor) (6 months)	No record	[22]
Coriander (*Coriandrum sativum* L.)	Linalool; λ-terpinene	Chickpea	65.5% inhibition at 2.5 μL/mL (vapor) (6 months)	No record	[22]
*Cymbopogon citratus*	Geranial; neral; myrcene	Tuberous root of *Asparagus racemosus*	78.4% inhibition at 500 ppm (*v*/*v*) (12 months)	100% inhibition at 500 ppm (*v*/*v*) on (12 months)	[32]
Dill (*Anethum graveolens* L.)	Carvone; limonene; apiol	Cherry tomato	88.9% inhibition at 120 μg/mL (vapor) (9 days)	No record	[37]
Fennel (*Foeniculum vulgare*)	Estragole; anethole	Tobacco leave	51.20–55.35% inhibition at 1.25 μL/mL (vapor) (6 months);	100% inhibition at 1.25 μL/mL (vapor) (6 months);	[38]
Fingerroot (*Boesenbergia rotunda*)	Nerol; L-camphor	In-shell peanut	No record	98.3% and 18.0% inhibition at 16% (*v*/*v*, in mineral oil) when applied via direct exposure and vapor exposure, respectively (10 days).	[39]
*Hedychium spicatum*	1,8-Cineole	Chickpea	72.0% inhibition at 2.5 μL/mL (vapor)	No record	[22]
Holy basil (*Ocimum sanctum*)	Eugenol; β-caryophyllene	Apocynaceae (*Rauvolfia serpentina* L., medicinal plant)	74.0% inhibition at 1 μL/mL (vapor) (6 months)	No record	[40]
*Hyptis suaveolens*	β -Caryophyllene; caryophyllene oxide; sabinene	Wheat	83.3% inhibition at 1.2 μL/mL (vapor) (12 months)	No record	[16]
Juniper (*Juniperus communis* L.)	α-Pinene	Polenta	No record	100% inhibition at 50 μg/g (14 days)	[24]
Lemongrass (*Cymbopogon citrati* [DC] Stapf.)	Neral; geranial	Bread	90% inhibition at 134.12 μL/L (vapor) (14 days)	100% inhibition at 125 μL/L (vapor) (14 days)	[23]
*Lippia alba*	Myrcene; neral; geranial	Green gram seed	92.5% inhibition at 80 μL/0.25 L (vapor) (6 months)	100% inhibition at 80 μL/0.25 L (vapor) (6 months)	[41]
*Litsea cubeba*	D-Limonene; (Z)-limonene oxide; (E)-limonene oxide	Licorice	100% inhibition at 5 μL/g (vapor) (20 days);	100% inhibition at 5 μL/g (vapor) (20 days);	[42]
Marjoram (*Origanum majorana* L.)	Terpinen-4-ol; cis-sabinene hydrate; *p*-cymene	Chickpea	67.9% inhibition at 3 μL/mL (vapor) (6 months)	No record	[22]
*Mentha spicata* L.	Carvone; limonene	Chickpea	52.2% inhibition at 1 μL/mL (vapor) (12 months);	100% inhibition at 1 μL/mL (vapor) (12 months);	[43]
*Michelia alba*	Linalool	Brown rice	100% inhibition at 300 μL/L (vapor) (12 weeks)	No record	[44]
Mint (*Mentha viridis*)	Menthone; carvone	Wheat; corn	100% inhibition at 200 mL/100 g in corn (21 days); 92% inhibition at 2% (*v*/*w*) in wheat (14 days)	100% inhibition at 300 mL/100 g in corn (21 days); >99% inhibition at 2% (v/w) in wheat (8 weeks)	[19,45]
Mountain thyme (*Hedeoma multiflora* Benth)	α-Terpinolene; *p*-cymene; carvacrol	Corn	100% inhibition at 500–2000 μg/g (dependent on water activity) (11 days);	100% inhibition at 1000 μg/g (11 days);	[6]
*Ocimum basilicum* L.	Methyl eugenol	Dry fruits (cashew nut, almond, grapes, chironji, groundnut, date palm, and coconut)	53.8–65.5% inhibition at 1 μg/mL (vapor) (6 months)	No record	[46]
Oregano (*Origanum vulgare* L.)	Carvacrol; linalool; 4-terpineol	Maize extract medium; bread; corn; soybean	90% inhibition at 820 → 1000 mg/L (depending on temperature and water activity) in maize extract medium (12 days); 90% inhibition at 319.85 μL/L (vapor) in bread (14 days); 100% inhibition with 5 μL EO in corn and soybean (disk diffusion assay, 5 days)	100% inhibition at >1000 mg/L in maize extract medium (12 days); 100% inhibition at 125 μL/L (vapor) in bread (14 days); >90% and 88.16% inhibition with 200 μL EO in 60 g corn and soybean, respectively (direct contact) (10 days)	[17,23,29]
Pine (*Pinus pinaster*)	β-Caryophyllene; β-selinene	In-shell peanut	No record	98.1% and 12.9% inhibition at 16% (*v*/*v*, in mineral oil) when applied via direct exposure and vapor exposure, respectively (10 days)	[39]
Poleo (*Lippia turbinate* var. integrifolia (griseb))	Peperitenone oxide; limonene	Corn	100% inhibition at 500–2000 μg/g (depending on water active) (11 days)	100% inhibition at 500–2000 μg/g (depending on water active) (11 days)	[6]
Rosewood (*Aniba rosaeodora*)	Linalool	In-shell peanut	No record	98.5% and 17.2% inhibition at 16% (*v*/*v*, in mineral oil) when applied via direct exposure and vapor exposure, respectively (10 days)	[39]
*Rosmarinus officinalis* L.	α-Pinene; 1, 8-cineole; camphor	Black pepper (*Piper nigrum*)	73.5% inhibition at 1.5 μL/mL (vapor) (6 months);	No record	[47]
*Styrax tonkinensis*	Benzoic acid; 6-phenyl-tetrahydro-naphthaline	In-shell peanut	No record	95.8% and 20.2% inhibition at 16% (*v*/*v*, in mineral oil) when applied via direct exposure and vapor exposure, respectively (10 days).	[39]
Summer savory (*Satureja hortensis*)	γ-terpinene; carvacrol; thymol	Tomato paste	59% inhibition at 500 ppm (2 months)	No record	[36]
Thyme (*Thymus vulgaris* L.)	Carvacrol; a-terpinolene; thymol; *p*-cymene; β-phellandrene; linalool	Bread; tomato paste; wheat; brown rice; white rice	90% inhibition at 474.2 μL/L (vapor) in bread (14 days); 87% inhibition at 500 ppm in tomato paste (2 months); 100% inhibition at 1% (*v*/*w*) in wheat (14 days)	100% inhibition at 250 μL/L (vapor) in bread (14 days); 100% inhibition at 1% (*v*/*w*) on wheat (8 weeks); 72.7% inhibition at 10 μg/mL (vapor) in brown rice; 18.0% inhibition at 10 μg/mL (vapor) in white rice	[19,23,36,48]
Thyme (*Zataria multiflora*)	Thymol; carvacrol	Iranian white cheese	89.0% inhibition at 600 ppm (up to 40 days)	92.9% inhibition at 600 ppm (up to 40 days)	[35]
*Thymus daenensis* Celak	Thymol; carvacrol	Pistachio	No record	100% inhibition with 25 ml of 9% (vol/vol) EO solution in 500 g pistachio (3 months)	[30]
Turmeric (*Curcuma longa* L.)	Tumerone; ar-turmerone; β-sesquiphellandrene; zingiberene; cycloisolongifolene	Corn	~90% inhibition at 4 μg/mL (5 days)	~93% inhibition at 4 μg/mL (5 days)	[49]
Vatica (*Vatica diospyroides* Symington)	Benzyl acetate	Corn	100% inhibition at 50 μL/L (vapor) (5 days)	100% inhibition at 50 μL/L (vapor) (5 days)	[34]
Ylang ylang (*Cananga odorata*)	Linalool; benzyl acetate; tetradecane; germacrene D	In-shell peanut	No record	96.4% and 25.1% inhibition at 16% (*v*/*v*, in mineral oil) when applied via direct exposure and vapor exposure, respectively (10 days).	[39]
*Zanthoxylum alatum*	Linalool; methyl cinnamate	Black pepper (*Piper nigrum*)	87.6% inhibition at 2.5 μL/mL (vapor) (6 months)	No record	[50]
*Zanthoxylum molle* Rehd	Limonene; terpinen-4-ol; 2-undecanone	Cherry tomato	91.7% inhibition at 0.2 μg/mL (vapor) (9 days)	No record	[51]
*Zingiber zerumbet*	α-Caryophyllene; zerumbone	Corn	100% inhibition at 200 ppm (15 days)	100% inhibition at 100 ppm (direct contact) (15 days)	[52]
*Ziziphora clinopodioides*	Pulegone; piperitenone; *p*-menth-3-en-8-ol	Corn	No record	99.8% inhibition at 6250 μg/mL (29 days)	[53]

## References

[1] Fountain J., Scully B., Ni X., Kemerait R., Lee D., Chen Z.-Y., Guo B. (2014). Environmental influences on maize-*Aspergillus flavus* interactions and aflatoxin production. Front. Microbiol..

[2] Amaike S., Keller N.P. (2011). *Aspergillus* *flavus*. Annu. Rev. Phytopathol..

[3] Baranyi N., Kocsubé S., Varga J. (2015). Aflatoxins: Climate change and biodegradation. Curr. Opin. Food Sci..

[4] Bartoletti M., Pascale R., Cricca M., Rinaldi M., Maccaro A., Bussini L., Fornaro G., Tonetti T., Pizzilli G., Francalanci E. (2021). Epidemiology of invasive pulmonary aspergillosis among intubated patients with COVID-19: A prospective study. Clin. Infect. Dis.

[5] Prakash B., Shukla R., Singh P., Kumar A., Mishra P.K., Dubey N.K. (2010). Efficacy of chemically characterized *Piper betle* L. essential oil against fungal and aflatoxin contamination of some edible commodities and its antioxidant activity. Int. J. Food Microbiol..

[6] Bluma R.V., Etcheverry M.G. (2008). Application of essential oils in maize grain: Impact on *Aspergillus* section *Flavi* growth parameters and aflatoxin accumulation. Food Microbiol..

[7] Lv X., Pan L., Wang J., Lu L., Yan W., Zhu Y., Xu Y., Guo M., Zhuang S. (2017). Effects of triazole fungicides on androgenic disruption and CYP3A4 enzyme activity. Environ. Pollut..

[8] Feng Y., Huang Y., Zhan H., Bhatt P., Chen S. (2020). An overview of strobilurin fungicide degradation: Current status and future perspective. Front. Microbiol..

[9] Rao J., Chen B., McClements D.J. (2019). Improving the efficacy of essential oils as antimicrobials in foods: Mechanisms of action. Annu. Rev. Food Sci. Technol..

[10] Khan F.A., Khan N.M., Ahmad S., Nasruddin, Aziz R., Ullah I., Almehmadi M., Allahyani M., Alsaiari A.A., Aljuaid A. (2022). Phytochemical profiling, antioxidant, antimicrobial and cholinesterase inhibitory effects of essential oils isolated from the leaves of *Artemisia scoparia* and *Artemisia absinthium*. Pharmaceuticals.

[11] Khan F.A., Khan S., Khan N.M., Khan H., Khan S., Ahmad S., Rehman N., Aziz R. (2021). Antimicrobial and antioxidant role of the aerial parts of *Aconitum violaceum*. J. Mex. Chem. Soc..

[12] Ali F., Jan A.K., Khan N.M., Ali R., Mukhtiar M., Khan S., Khan S.A., Aziz R. (2018). Selective biological activities and phytochemical profiling of two wild plant species, *Teucrium polium* and *Capsicum annum* from Sheringal, Pakistan. Chiang Mai J. Sci..

[13] Khan H., Ali F., Khan N.M., Shah A., Rahman S.U. (2016). GC-MS Analysis of fixed oil from *Nelumbo nucifera* Gaertn seeds: Evaluation of antimicrobial, antileishmanial and urease inhibitory activities. J. Chem. Soc. Pak..

[14] Hu Z.Y., Yuan K., Zhou Q., Lu C., Du L.H., Liu F. (2021). Mechanism of antifungal activity of *Perilla frutescens* essential oil against *Aspergillus flavus* by transcriptomic analysis. Food Control.

[15] Oliveira R.C., Carvajal-Moreno M., Correa B., Rojo-Callejas F. (2020). Cellular, physiological and molecular approaches to investigate the antifungal and anti-aflatoxigenic effects of thyme essential oil on *Aspergillus flavus*. Food Chem..

[16] Prakash B., Dubey N.K. (2011). Evaluation of chemically characterised essential oils of *Coleus aromaticus*, *Hyptis suaveolens* and *Ageratum conyzoides* against storage fungi and aflatoxin contamination of food commodities. Int. J. Food Sci. Technol..

[17] Esper R.H., Goncalez E., Marques M.O., Felicio R.C., Felicio J.D. (2014). Potential of essential oils for protection of grains contaminated by aflatoxin produced by *Aspergillus flavus*. Front. Microbiol..

[18] Kedia A., Prakash B., Mishra P.K., Dwivedy A.K., Dubey N. (2015). *Trachyspermum ammi* L. essential oil as plant based preservative in food system. Ind. Crops Prod..

[19] Soliman K.M., Badeaa R. (2002). Effect of oil extracted from some medicinal plants on different mycotoxigenic fungi. Food Chem. Toxicol..

[20] Prakash B., Mishra P.K., Kedia A., Dubey N. (2014). Antifungal, antiaflatoxin and antioxidant potential of chemically characterized *Boswellia carterii* Birdw essential oil and its *in vivo* practical applicability in preservation of *Piper nigrum* L. fruits. LWT Food Sci. Technol..

[21] Venkatesh H.N., Sudharshana T.N., Abhishek R.U., Thippeswamy S., Manjunath K., Mohana D.C. (2017). Antifungal and antimycotoxigenic properties of chemically characterised essential oil of *Boswellia serrata* Roxb. ex Colebr. Int. J. Food Prop..

[22] Prakash B., Singh P., Kedia A., Dubey N. (2012). Assessment of some essential oils as food preservatives based on antifungal, antiaflatoxin, antioxidant activities and in vivo efficacy in food system. Food Res. Int..

[23] Císarová M., Hleba L., Medo J., Tančinová D., Mašková Z., Čuboň J., Kováčik A., Foltinová D., Božik M., Klouček P. (2020). The *in vitro* and *in situ* effect of selected essential oils in vapour phase against bread spoilage toxicogenic aspergilli. Food Control.

[24] Kocić-Tanackov S., Dimić G., Jakšić S., Mojović L., Djukić-Vuković A., Mladenović D., Pejin J. (2019). Effects of caraway and juniper essential oils on aflatoxigenic fungi growth and aflatoxins secretion in polenta. J. Food Process. Preserv..

[25] Kazemi M. (2015). Effect of *Carum copticum* essential oil on growth and aflatoxin formation by *Aspergillus* strains. Nat. Prod. Res..

[26] Pandey A.K., Singh P., Palni U.T., Tripathi N. (2013). Application of *Chenopodium ambrosioides* Linn. essential oil as botanical fungicide for the management of fungal deterioration in pulses. Biol. Agric. Hortic..

[27] Tian J., Ban X., Zeng H., He J., Huang B., Wang Y. (2011). Chemical composition and antifungal activity of essential oil from *Cicuta virosa* L. var. latisecta Celak. Int. J. Food Microbiol..

[28] Prakash B., Singh P., Yadav S., Singh S.C., Dubey N.K. (2013). Safety profile assessment and efficacy of chemically characterized *Cinnamomum glaucescens* essential oil against storage fungi, insect, aflatoxin secretion and as antioxidant. Food Chem. Toxicol..

[29] Gómez J.V., Tarazona A., Mateo-Castro R., Gimeno-Adelantado J.V., Jiménez M., Mateo E.M. (2018). Selected plant essential oils and their main active components, a promising approach to inhibit aflatoxigenic fungi and aflatoxin production in food. Food Addit. Contam. Chem. Anal. Control Expo. Risk Assess..

[30] Khorasani S., Azizi M.H., Barzegar M., Hamidi-Esfahani Z., Kalbasi-Ashtari A. (2017). Inhibitory effects of cinnamon, clove and celak extracts on growth of *Aspergillus flavus* and its aflatoxins after spraying on pistachio nuts before cold storage. J. Food Saf..

[31] Kiran S., Kujur A., Prakash B. (2016). Assessment of preservative potential of *Cinnamomum zeylanicum* Blume essential oil against food borne molds, aflatoxin B_1_ synthesis, its functional properties and mode of action. Innov. Food Sci. Emerg. Technol..

[32] Singh P., Shukla R., Kumar A., Prakash B., Singh S., Dubey N.K. (2010). Effect of *Citrus reticulata* and *Cymbopogon citratus* essential oils on *Aspergillus flavus* growth and aflatoxin production on *Asparagus racemosus*. Mycopathologia.

[33] Pandey A.K., Palni U.T., Tripathi N.N. (2013). Evaluation of *Clausena pentaphylla* (Roxb.) DC oil as a fungitoxicant against storage mycoflora of pigeon pea seeds. J. Sci. Food Agric..

[34] Boukaew S., Prasertsan P., Sattayasamitsathit S. (2017). Evaluation of antifungal activity of essential oils against aflatoxigenic *Aspergillus flavus* and their allelopathic activity from fumigation to protect maize seeds during storage. Ind. Crops Prod..

[35] Moosavi-Nasab M., Jamalian J., Heshmati H., Haghighi-Manesh S. (2018). The inhibitory potential of *Zataria multiflora* and *Syzygium aromaticum* essential oil on growth and aflatoxin production by *Aspergillus flavus* in culture media and Iranian white cheese. Food Sci. Nutr..

[36] Omidbeygi M., Barzegar M., Hamidi Z., Naghdibadi H. (2007). Antifungal activity of thyme, summer savory and clove essential oils against *Aspergillus flavus* in liquid medium and tomato paste. Food Control.

[37] Tian J., Ban X., Zeng H., Huang B., He J., Wang Y. (2011). *In vitro* and *in vivo* activity of essential oil from dill (*Anethum graveolens* L.) against fungal spoilage of cherry tomatoes. Food Control.

[38] Kedia A., Dwivedy A.K., Pandey A.K., Kumar R.R., Regmi P., Dubey N.K. (2015). Efficacy of chemically characterized *Foeniculum vulgare* Mill seed essential oil in protection of raw tobacco leaves during storage against fungal and aflatoxin contamination. J. Appl. Microbiol..

[39] Jantapan K., Poapolathep A., Imsilp K., Poapolathep S., Tanhan P., Kumagai S., Jermnak U. (2017). Inhibitory Effects of Thai essential oils on potentially aflatoxigenic *Aspergillus parasiticus* and *Aspergillus flavus*. Biocontrol. Sci..

[40] Kumar A., Dubey N.K., Srivastava S. (2013). Antifungal evaluation of *Ocimum sanctum* essential oil against fungal deterioration of raw materials of *Rauvolfia serpentina* during storage. Ind. Crops Prod..

[41] Pandey A.K., Sonker N., Singh P. (2016). Efficacy of some essential oils against *Aspergillus flavus* with special reference to *Lippia alba* oil an inhibitor of fungal proliferation and aflatoxin B_1_ production in green gram seeds during storage. J. Food Sci..

[42] Li Y., Kong W., Li M., Liu H., Zhao X., Yang S., Yang M. (2016). *Litsea cubeba* essential oil as the potential natural fumigant: Inhibition of *Aspergillus flavus* and AFB_1_ production in licorice. Ind. Crops Prod..

[43] Kedia A., Dwivedy A.K., Jha D.K., Dubey N.K. (2016). Efficacy of *Mentha spicata* essential oil in suppression of *Aspergillus flavus* and aflatoxin contamination in chickpea with particular emphasis to mode of antifungal action. Protoplasma.

[44] Songsamoe S., Matan N., Matan N. (2017). Antifungal activity of *Michelia alba* oil in the vapor phase and the synergistic effect of major essential oil components against *Aspergillus flavus* on brown rice. Food Control.

[45] Gibriel Y., Hamza A., Gibriel A., Mohsen S. (2011). In Vivo effect of mint (*Mentha viridis*) essential oil on growth and aflatoxin production by *Aspergillus flavus* isolated from stored corn. J. Food Saf..

[46] Kumar A., Shukla R., Singh P., Prakash B., Dubey N.K. (2011). Chemical composition of *Ocimum basilicum* L. essential oil and its efficacy as a preservative against fungal and aflatoxin contamination of dry fruits. Int. J. Food Sci. Technol..

[47] Prakash B., Kedia A., Mishra P.K., Dwivedy A.K., Dubey N.K. (2015). Assessment of chemically characterised *Rosmarinus officinalis* L. essential oil and its major compounds as plant-based preservative in food system based on their efficacy against food-borne moulds and aflatoxin secretion and as antioxidant. Int. J. Food Sci. Technol..

[48] Tian F., Lee S.Y., Chun H.S. (2019). Comparison of the antifungal and antiaflatoxigenic potential of liquid and vapor phase of *Thymus vulgaris* essential oil against *Aspergillus flavus*. J. Food Prot..

[49] Hu Y., Zhang J., Kong W., Zhao G., Yang M. (2017). Mechanisms of antifungal and anti-aflatoxigenic properties of essential oil derived from turmeric (*Curcuma longa* L.) on *Aspergillus flavus*. Food Chem..

[50] Prakash B., Singh P., Mishra P.K., Dubey N.K. (2012). Safety assessment of *Zanthoxylum alatum* Roxb. essential oil, its antifungal, antiaflatoxin, antioxidant activity and efficacy as antimicrobial in preservation of *Piper nigrum* L. fruits. Int. J. Food Microbiol..

[51] Tian J., Zeng X., Feng Z., Miao X., Peng X., Wang Y. (2014). *Zanthoxylum molle* Rehd. essential oil as a potential natural preservative in management of *Aspergillus flavus*. Ind. Crops Prod..

[52] Madegowda B.H., Rameshwaran P., Nagaraju N.P., Murthy P.S. (2016). *In-vitro* Mycological activity of essential oil from *Zingiber zerumbet* rhizomes. J. Essent. Oil Res..

[53] Moghadam H.D., Sani A.M., Sangatash M.M. (2016). Antifungal activity of essential oil of *Ziziphora clinopodioides* and the inhibition of aflatoxin B_1_ production in maize grain. Toxicol. Ind. Health.

[54] Hu Y., Kong W., Yang X., Xie L., Wen J., Yang M. (2014). GC-MS combined with chemometric techniques for the quality control and original discrimination of *Curcumae longae* rhizome: Analysis of essential oils. J. Sep. Sci..

[55] Ojeda-Amador R.M., Fregapane G., Salvador M.D. (2020). Influence of cultivar and technological conditions on the volatile profile of virgin pistachio oils. Food Chem..

[56] Ben Arfa A., Combes S., Preziosi-Belloy L., Gontard N., Chalier P. (2006). Antimicrobial activity of carvacrol related to its chemical structure. Lett. Appl. Microbiol..

[57] Tian F., Woo S.Y., Lee S.Y., Park S.B., Im J.H., Chun H.S. (2023). Plant-based natural flavonoids show strong inhibition of aflatoxin production and related gene expressions correlated with chemical structure. Food Microbiol..

[58] Cox S.D., Mann C.M., Markham J.L., Gustafson J.E., Warmington J.R., Wyllie S.G. (2001). Determining the antimicrobial actions of tea tree oil. Molecules.

[59] Dorman H.J., Deans S.G. (2000). Antimicrobial agents from plants: Antibacterial activity of plant volatile oils. J. Appl. Microbiol..

[60] Burt S. (2004). Essential oils: Their antibacterial properties and potential applications in foods—A review. Int. J. Food Microbiol..

[61] Passone M.A., Girardi N.S., Etcheverry M. (2013). Antifungal and antiaflatoxigenic activity by vapor contact of three essential oils, and effects of environmental factors on their efficacy. LWT Food Sci. Technol..

[62] Skandamis P.N., Nychas G.J. (2000). Development and evaluation of a model predicting the survival of *Escherichia coli* O157:H7 NCTC 12900 in homemade eggplant salad at various temperatures, pHs, and oregano essential oil concentrations. Appl. Environ. Microbiol..

[63] Rota M.C., Herrera A., Martínez R.M., Sotomayor J.A., Jordán M.J. (2008). Antimicrobial activity and chemical composition of *Thymus vulgaris*, *Thymus zygis* and *Thymus hyemalis* essential oils. Food Control.

[64] Sarrazin S.L., Oliveira R.B., Barata L.E., Mourao R.H. (2012). Chemical composition and antimicrobial activity of the essential oil of *Lippia grandis* Schauer (Verbenaceae) from the western Amazon. Food Chem..

[65] Stević T., Berić T., Šavikin K., Soković M., Gođevac D., Dimkić I., Stanković S. (2014). Antifungal activity of selected essential oils against fungi isolated from medicinal plant. Ind. Crops Prod..

[66] Hossain F., Follett P., Dang Vu K., Harich M., Salmieri S., Lacroix M. (2016). Evidence for synergistic activity of plant-derived essential oils against fungal pathogens of food. Food Microbiol..

[67] Goni P., López P., Sánchez C., Gómez-Lus R., Becerril R., Nerín C. (2009). Antimicrobial activity in the vapour phase of a combination of cinnamon and clove essential oils. Food Chem..

[68] Lamberts L., De Bie E., Vandeputte G.E., Veraverbeke W.S., Derycke V., De Man W., Delcour J.A. (2007). Effect of milling on colour and nutritional properties of rice. Food Chem..

[69] Tang J.D., Ciaramitaro T., Tomaso-Peterson M., Diehl S.V. Activity of two strobilurin fungicides against three species of decay fungi in agar plate tests. Proceedings of the International Research Group on Wood Protection, Section 3, Wood Protecting Chemicals: Paper Prepared for the IRG48 Scientific Conference on Wood Protection.

[70] Tian F., Lee S.Y., Woo S.Y., Choi H.Y., Park S.B., Chun H.S. (2021). Effect of plant-based compounds on the antifungal and antiaflatoxigenic efficiency of strobilurins against *Aspergillus flavus*. J. Hazard. Mater..

[71] Khan M.S.A., Ahmad I. (2011). Antifungal activity of essential oils and their synergy with fluconazole against drug-resistant strains of *Aspergillus fumigatus* and *Trichophyton rubrum*. Appl. Microbiol. Biotechnol..

[72] Belofsky G., Kolaczkowski M., Adams E., Schreiber J., Eisenberg V., Coleman C.M., Zou Y., Ferreira D. (2013). Fungal ABC transporter-associated activity of isoflavonoids from the root extract of *Dalea formosa*. J. Nat. Prod..

[73] Fukuda I., Ashida H., Watson R.R., Preedy V.R., Zibadi S. (2014). Modulation of drug-metabolizing enzymes and transporters by polyphenols as an anticarcinogenic effect. Polyphenols in Human Health and Disease.

[74] Ziberna L., Fornasaro S., Čvorović J., Tramer F., Passamonti S., Watson R.R., Preedy V.R., Zibadi S. (2014). Bioavailability of flavonoids: The role of cell membrane transporters. Polyphenols in Human Health and Disease.

[75] Bowman S.M., Free S.J. (2006). The structure and synthesis of the fungal cell wall. Bioessays.

[76] Li Q., Zhao Y., Zhu X.M., Xie Y.L. (2021). Antifungal efficacy of paeonol on *Aspergillus flavus* and its mode of action on cell walls and cell membranes. LWT-Food Sci. Technol..

[77] Belewa V., Baijnath H., Frost C., Somai B.M. (2017). *Tulbaghia violacea* Harv. plant extract affects cell wall synthesis in *Aspergillus flavus*. J. Appl. Microbiol..

[78] da Silva Bomfim N., Nakassugi L.P., Faggion Pinheiro Oliveira J., Kohiyama C.Y., Mossini S.A., Grespan R., Nerilo S.B., Mallmann C.A., Alves Abreu Filho B., Machinski M. (2015). Antifungal activity and inhibition of fumonisin production by *Rosmarinus officinalis* L. essential oil in *Fusarium verticillioides* (Sacc.) Nirenberg. Food Chem..

[79] Gow N.A.R., Latge J.P., Munro C.A. (2017). The fungal cell wall: Structure, biosynthesis, and function. Microbiol. Spectr..

[80] Marei G.I.K., Rasoul M.A.A., Abdelgaleil S.A. (2012). Comparative antifungal activities and biochemical effects of monoterpenes on plant pathogenic fungi. Pestic. Biochem. Physiol..

[81] Bang K.H., Lee D.W., Park H.M., Rhee Y.H. (2000). Inhibition of fungal cell wall synthesizing enzymes by trans-cinnamaldehyde. Biosci. Biotechnol. Biochem..

[82] Bakkali F., Averbeck S., Averbeck D., Idaomar M. (2008). Biological effects of essential oils—A review. Food Chem. Toxicol..

[83] Di Pasqua R., Betts G., Hoskins N., Edwards M., Ercolini D., Mauriello G. (2007). Membrane toxicity of antimicrobial compounds from essential oils. J. Agric. Food Chem..

[84] Pauli A. (2006). Anticandidal low molecular compounds from higher plants with special reference to compounds from essential oils. Med. Res. Rev..

[85] Dwivedy A.K., Kumar M., Upadhyay N., Prakash B., Dubey N.K. (2016). Plant essential oils against food borne fungi and mycotoxins. Curr. Opin. Food Sci..

[86] Ghannoum M.A., Rice L.B. (1999). Antifungal agents: Mode of action, mechanisms of resistance, and correlation of these mechanisms with bacterial resistance. Clin. Microbiol. Rev..

[87] Freires Ide A., Murata R.M., Furletti V.F., Sartoratto A., Alencar S.M., Figueira G.M., de Oliveira Rodrigues J.A., Duarte M.C., Rosalen P.L. (2014). *Coriandrum sativum* L. (Coriander) essential oil: Antifungal activity and mode of action on *Candida* spp., and molecular targets affected in human whole-genome expression. PLoS ONE.

[88] Tian J., Huang B., Luo X., Zeng H., Ban X., He J., Wang Y. (2012). The control of *Aspergillus flavus* with *Cinnamomum jensenianum* Hand.-Mazz essential oil and its potential use as a food preservative. Food Chem..

[89] Kohiyama C.Y., Ribeiro M.M.Y., Mossini S.A.G., Bando E., da Silva Bomfim N., Nerilo S.B., Rocha G.H.O., Grespan R., Mikcha J.M.G., Machinski Jr M. (2015). Antifungal properties and inhibitory effects upon aflatoxin production of *Thymus vulgaris* L. by *Aspergillus flavus* Link. Food Chem..

[90] Tian J., Ban X., Zeng H., He J., Chen Y., Wang Y. (2012). The mechanism of antifungal action of essential oil from dill (*Anethum graveolens* L.) on *Aspergillus flavus*. PLoS ONE.

[91] OuYang Q., Tao N., Jing G. (2016). Transcriptional profiling analysis of *Penicillium digitatum*, the causal agent of citrus green mold, unravels an inhibited ergosterol biosynthesis pathway in response to citral. BMC Genom..

[92] Ultee A., Bennik M., Moezelaar R. (2002). The phenolic hydroxyl group of carvacrol is essential for action against the food-borne pathogen *Bacillus cereus*. Appl. Environ. Microbiol..

[93] Trombetta D., Castelli F., Sarpietro M.G., Venuti V., Cristani M., Daniele C., Saija A., Mazzanti G., Bisignano G. (2005). Mechanisms of antibacterial action of three monoterpenes. Antimicrob. Agents Chemother..

[94] Dagley M.J., Gentle I.E., Beilharz T.H., Pettolino F.A., Djordjevic J.T., Lo T.L., Uwamahoro N., Rupasinghe T., Tull D.L., McConville M. (2011). Cell wall integrity is linked to mitochondria and phospholipid homeostasis in *Candida albicans* through the activity of the post-transcriptional regulator Ccr4-Pop2. Mol. Microbiol..

[95] Ma W., Zhao L., Zhao W., Xie Y. (2019). ( E)-2-Hexenal, as a potential natural antifungal compound, inhibits *Aspergillus flavus* spore germination by disrupting mitochondrial energy metabolism. J. Agric. Food Chem..

[96] Ma W.B., Zhao L.L., Johnson E.T., Xie Y.L., Zhang M.M. (2022). Natural food flavour (E)-2-hexenal, a potential antifungal agent, induces mitochondria-mediated apoptosis in *Aspergillus flavus* conidia via a ROS-dependent pathway. Int. J. Food Microbiol..

[97] Zhang W., Li B., Lv Y., Wei S., Zhang S., Hu Y. (2022). Transcriptomic analysis shows the antifungal mechanism of honokiol against *Aspergillus flavus*. Int. J. Food Microbiol..

[98] Li Y., Shao X., Xu J., Wei Y., Xu F., Wang H. (2017). Tea tree oil exhibits antifungal activity against *Botrytis cinerea* by affecting mitochondria. Food Chem..

[99] Zheng S., Jing G., Wang X., Ouyang Q., Jia L., Tao N. (2015). Citral exerts its antifungal activity against *Penicillium digitatum* by affecting the mitochondrial morphology and function. Food Chem..

[100] Rao A., Zhang Y., Muend S., Rao R. (2010). Mechanism of antifungal activity of terpenoid phenols resembles calcium stress and inhibition of the TOR pathway. Antimicrob. Agents Chemother..

[101] Pinto E., Hrimpeng K., Lopes G., Vaz S., Goncalves M.J., Cavaleiro C., Salgueiro L. (2013). Antifungal activity of *Ferulago capillaris* essential oil against *Candida*, *Cryptococcus*, *Aspergillus* and dermatophyte species. Eur. J. Clin. Microbiol. Infect. Dis..

[102] Loi M., Paciolla C., Logrieco A.F., Mule G. (2020). Plant bioactive compounds in pre- and postharvest management for aflatoxins reduction. Front. Microbiol..

[103] Chaudhari A.K., Dwivedy A.K., Singh V.K., Das S., Singh A., Dubey N.K. (2019). Essential oils and their bioactive compounds as green preservatives against fungal and mycotoxin contamination of food commodities with special reference to their nanoencapsulation. Environ. Sci. Pollut. Res..

[104] Calvo A.M., Wilson R.A., Bok J.W., Keller N.P. (2002). Relationship between secondary metabolism and fungal development. Microbiol. Mol. Biol. Rev..

[105] Osiewacz H.D. (2011). Mitochondrial quality control in aging and lifespan control of the fungal aging model *Podospora anserina*. Biochem. Soc. Trans..

[106] Molnár A.P., Nemeth Z., Fekete E., Flipphi M., Keller N.P., Karaffa L. (2018). Analysis of the relationship between alternative respiration and sterigmatocystin formation in *Aspergillus nidulans*. Toxins.

[107] Bluma R., Amaiden M.R., Daghero J., Etcheverry M. (2008). Control of *Aspergillus* section *Flavi* growth and aflatoxin accumulation by plant essential oils. J. Appl. Microbiol..

[108] Scarpari M., Punelli M., Scala V., Zaccaria M., Nobili C., Ludovici M., Camera E., Fabbri A.A., Reverberi M., Fanelli C. (2014). Lipids in *Aspergillus flavus*-maize interaction. Front. Microbiol..

[109] Zaccaria M., Ludovici M., Sanzani S.M., Ippolito A., Cigliano R.A., Sanseverino W., Scarpari M., Scala V., Fanelli C., Reverberi M. (2015). Menadione-induced oxidative stress re-shapes the oxylipin profile of *Aspergillus flavus* and its lifestyle. Toxins.

[110] Roze L.V., Laivenieks M., Hong S.Y., Wee J., Wong S.S., Vanos B., Awad D., Ehrlich K.C., Linz J.E. (2015). Aflatoxin biosynthesis is a novel source of reactive oxygen species-a potential redox signal to initiate resistance to oxidative stress?. Toxins.

[111] Umesha S., Manukumar H.M., Chandrasekhar B., Shivakumara P., Shiva Kumar J., Raghava S., Avinash P., Shirin M., Bharathi T.R., Rajini S.B. (2017). Aflatoxins and food pathogens: Impact of biologically active aflatoxins and their control strategies. J. Sci. Food Agric..

[112] Kim J.H., Campbell B.C., Yu J., Mahoney N., Chan K.L., Molyneux R.J., Bhatnagar D., Cleveland T.E. (2005). Examination of fungal stress response genes using *Saccharomyces cerevisiae* as a model system: Targeting genes affecting aflatoxin biosynthesis by *Aspergillus flavus* Link. Appl. Microbiol. Biotechnol..

[113] Jahanshiri Z., Shams-Ghahfarokhi M., Allameh A., Razzaghi-Abyaneh M. (2015). Inhibitory effect of eugenol on aflatoxin B_1_ production in *Aspergillus parasiticus* by downregulating the expression of major genes in the toxin biosynthetic pathway. World J. Microbiol. Biotechnol..

[114] Moon Y.S., Lee H.S., Lee S.E. (2018). Inhibitory effects of three monoterpenes from ginger essential oil on growth and aflatoxin production of *Aspergillus flavus* and their gene regulation in aflatoxin biosynthesis. Appl. Biol. Chem..

[115] Lv C., Wang P., Ma L., Zheng M., Liu Y., Xing F. (2018). Large-scale comparative analysis of eugenol-induced/repressed genes expression in *Aspergillus flavus* using RNA-seq. Front. Microbiol..

[116] Prakash B., Shukla R., Singh P., Mishra P.K., Dubey N.K., Kharwar R.N. (2011). Efficacy of chemically characterized *Ocimum gratissimum* L. essential oil as an antioxidant and a safe plant based antimicrobial against fungal and aflatoxin B_1_ contamination of spices. Food Res. Int..

[117] Shukla R., Singh P., Prakash B., Dubey N.K. (2013). Efficacy of *Acorus calamus* L. essential oil as a safe plant-based antioxidant, aflatoxin B_1_ suppressor and broad spectrum antimicrobial against food-infesting fungi. Int. J. Food Sci. Technol..

[118] Singh H.P., Mittal S., Kaur S., Batish D.R., Kohli R.K. (2009). Chemical composition and antioxidant activity of essential oil from residues of *Artemisia scoparia*. Food Chem..

[119] Ahmad-Qasem M.H., Canovas J., Barrajon-Catalan E., Micol V., Carcel J.A., Garcia-Perez J.V. (2013). Kinetic and compositional study of phenolic extraction from olive leaves (var. Serrana) by using power ultrasound. Innov. Food Sci. Emerg. Technol..

[120] Barba F.J., Terefe N.S., Buckow R., Knorr D., Orlien V. (2015). New opportunities and perspectives of high pressure treatment to improve health and safety attributes of foods. A review. Food Res. Int..

[121] Giacometti J., Kovacevic D.B., Putnik P., Gabric D., Bilusic T., Kresic G., Stulic V., Barba F.J., Chemat F., Barbosa-Canovas G. (2018). Extraction of bioactive compounds and essential oils from mediterranean herbs by conventional and green innovative techniques: A review. Food Res. Int..

[122] Singh A., Dwivedy A.K., Singh V.K., Upadhyay N., Chaudhari A.K., Das S., Dubey N.K. (2019). Essential oils based formulations as safe preservatives for stored plant masticatories against fungal and mycotoxin contamination: A review. Biocatal. Agric. Biotechnol..

[123] El Asbahani A., Miladi K., Badri W., Sala M., Ait Addi E.H., Casabianca H., El Mousadik A., Hartmann D., Jilale A., Renaud F.N. (2015). Essential oils: From extraction to encapsulation. Int. J. Pharm.

[124] Moretti M.D., Sanna-Passino G., Demontis S., Bazzoni E. (2002). Essential oil formulations useful as a new tool for insect pest control. AAPS PharmSciTech.

[125] Das S., Singh V.K., Dwivedy A.K., Chaudhari A.K., Upadhyay N., Singh A., Deepika, Dubey N.K. (2020). Fabrication, characterization and practical efficacy of *Myristica fragrans* essential oil nanoemulsion delivery system against postharvest biodeterioration. Ecotoxicol. Environ. Saf..

[126] Gundewadi G., Sarkar D.J., Rudra S.G., Singh D. (2018). Preparation of basil oil nanoemulsion using *Sapindus mukorossi* pericarp extract: Physico-chemical properties and antifungal activity against food spoilage pathogens. Ind. Crops Prod..

[127] Dávila-Rodríguez M., López-Malo A., Palou E., Ramírez-Corona N., Jiménez-Munguía M.T. (2019). Antimicrobial activity of nanoemulsions of cinnamon, rosemary, and oregano essential oils on fresh celery. LWT.

[128] Wan J., Zhong S., Schwarz P., Chen B., Rao J. (2019). Physical properties, antifungal and mycotoxin inhibitory activities of five essential oil nanoemulsions: Impact of oil compositions and processing parameters. Food Chem..

[129] Sies H., Jones D.P. (2020). Reactive oxygen species (ROS) as pleiotropic physiological signalling agents. Nat. Rev. Mol. Cell Biol..

